# Past, present, and future of epigenetics applied to livestock breeding

**DOI:** 10.3389/fgene.2015.00305

**Published:** 2015-09-28

**Authors:** Oscar González-Recio, Miguel A. Toro, Alex Bach

**Affiliations:** ^1^Departamento de Mejora Genética Animal, Instituto Nacional de Investigación y Tecnología Agraria y Alimentaria, MadridSpain; ^2^Departamento de Producción Animal, Escuela Técnica Superior de Ingenieros Agrónomos, Universidad Politécnica de MadridMadrid, Spain; ^3^Department of Ruminant Production, Institució Catalana de Recerca i Estudis Avançats – Institut de Recerca i Tecnologia Agroalimentàries, Caldes de MontbuiSpain

**Keywords:** epigenetics, animal breeding, population genomics, sustainability, breeding programs

## Abstract

This article reviews the concept of Lamarckian inheritance and the use of the term epigenetics in the field of animal genetics. *Epigenetics* was first coined by Conrad Hal Waddington (1905–1975), who derived the term from the Aristotelian word *epigenesis*. There exists some controversy around the word epigenetics and its broad definition. It includes any modification of the expression of genes due to factors other than mutation in the DNA sequence. This involves DNA methylation, post-translational modification of histones, but also linked to regulation of gene expression by non-coding RNAs, genome instabilities or any other force that could modify a phenotype. There is little evidence of the existence of transgenerational epigenetic inheritance in mammals, which may commonly be confounded with environmental forces acting simultaneously on an individual, her developing fetus and the germ cell lines of the latter, although it could have an important role in the cellular energetic status of cells. Finally, we review some of the scarce literature on the use of epigenetics in animal breeding programs.

## The old Ideas

Jean–Baptiste de Lamarck (1806) (1744–1829) first proposed a clearly evolutionary theory in his *Philosophie Zoologique*. He questioned the fixity of species, but his theory was quite confusing. He proposed that primitive organisms were generated spontaneously and then changed progressively along a so-called ‘Chain of Being’. In fact new forms were constantly generated and ascending toward greater complexity. His theory had little influence because it was rejected by the major figure in natural sciences at the time, France Georges Cuvier (1769–1832). Darwin (1868) also rejected Lamarck’s belief that organisms had an inherent drive to evolve in higher and more complex forms although he accepted that acquired characters can be inherited. However, in the late 19th century a ‘neo-Lamarckian’ school emphasized the inheritance of acquired characteristics in evolution. Although this idea was not original to Lamarck, it is actually associated under the umbrella of ‘Lamarckism’. It should be taken into consideration because it constitutes an alternative mechanism of evolution, and can be summarized as follows. During their life, organisms adapt to their environment with phenotypic changes allowing better survival. For example, people living in high altitudes produce more red blood cells, and similarly, animals develop immunity against most infective agents to which they are exposed. The example Lamarck gave was the blacksmith that develops arm muscles as a consequence of his work. If these changes that occur in an individual were transmitted in some way to a descendent, it would contribute to the evolution of new and improved adaptations. Another typical example from Lamarck was the giraffe, whose ancestor was thought to have acquired a long neck by stretching to reach upper leaves and to have transmitted it to their offspring. Darwin gave greater importance to this mechanism in a later edition of the ‘On the Origin of Species (Darwin, 1859)’ although he never thought it was as important as natural selection.

The Lamarckian theory of inheritance was explicitly rejected by the German biologist August Weissmann (1834–1914). He made the distinction between soma and germen. From the fertilized egg there are two independent processes of cell division, one leading to the body or soma and the other, the germ line, leading to the gametes that constitute the starting point of the next generation. The soma inevitably dies when the organism dies, but reproduction is concentrated in the germ line, which is, therefore, potentially immortal. Hence, selection between cell lines is possible. As it happens in cancer, a cell can mutate and out-reproduce other cells but, fortunately, this ‘adaptation’ will not be passed on to offspring. The point that Weissmann made is that the germ line is independent of changes in the soma, and therefore acquired characters cannot be inherited. Weissmann could not foresee how the strong muscles of the blacksmith could influence its sperm so that its offspring would in turn develop strong muscles. We should not dismiss the great insight of Weissmann because he realized that it is a problem of transmitting information: the body can provide nutrients and energy to the gametes but not information. In Weismann’s (1904, p.107) words: ‘if one came across a case of the inheritance of an acquired character, it would be as if a man sent a telegram to China and it arrived translated into Chinese’.

Today, we express the Weissmann argument as the Central Dogma of molecular biology (DNA makes RNA, and RNA makes protein), put forward in 1947 and presented formally by Crick in 1958: ‘Once information has passed into protein, it cannot get out again’. At that time, this idea was not supported by reasonable evidence, but now it can be justified in two ways. First, because the genetic code is degenerate; i.e., several triplets codify for each amino acid, and hence it is not possible to reconstruct the exact DNA sequence from protein sequence. Second, because a back-translation from protein to DNA sequence would require complex machinery, and such machinery has not yet been found. If a protein with a new amino acid sequence is present in a cell, it cannot cause the production of DNA with the corresponding base sequence.

However, the Central Dogma has crucial implications for evolution and animal breeding. It implies that artificial or natural selection requires changes in the frequency of the allelic distribution and that novelties can only arise through random and non-adaptive changes in the DNA sequence, i.e., mutations. Environmental factors, both at the cellular levels (i.e., the cytoplasm) and more macroscopic ones such as changes in maternal milk composition or allowance would affect the offspring development, but they would not alter the DNA sequence in gametes and therefore would not exert permanent effects.

Notice again that the Central Dogma is not a logical necessity but a fact of the inheritance system and therefore, as commonly happens in biology, one might expect some exceptions. Some of these exceptions can be found in many genetics text books and are commonly attributed to Lamarck. Perhaps the most popular are: (i) changes in gene amplification in flax (Linum): if flax plants are treated with high levels of fertilizer their morphology changes because some DNA sequences are repeated in a high number of copies. This change persists in absence of treatment for a number of generations (Durrant, 1962). (ii) Cortical inheritance in ciliates: in ciliate protozoa, the cell membrane surface presents a complex pattern of cilia. If the pattern of an individual is changed either accidentally or by physical manipulation, the new pattern is transmitted during many sexual and asexual generations (Sonneborn, 1937). And, (iii) morphology in water fleas of the crustacean Daphnia: when females are exposed to chemical signals associated with the presence of predators they develop a protective helmet that makes them less vulnerable to attack. Their offspring maintain the same defense, even when not exposed to predators (Laforsch and Tollrian, 2004).

There are two additional situations where no controversy exists. When cells of higher organisms are differentiated, these differences between cells are hereditary, but this differentiation is absent in the germ line. On the other hand cultural inheritance is typically Lamarckian.

During the last two centuries, Lamarckian ideas found temporal refuge in different disciplines. For example, the prevailing view in microbiology until the 1930s was that bacteria are different from plants and animals as they adapt and are modified by direct influence of the environment. This view changed with the classical work of the founders of molecular biology: Luria, Avery, and Delbruck. In immunology, Steele et al. (1998) claimed that environment could make the immune system to change its DNA structure, and these changes could be transmitted to the offspring, assertions that have yet to be confirmed.

Lastly, we should recall, that the reaction against Lamarckism was perhaps overly aggressive after it was adopted as political dogma in Stalin’s USSR during the 1940s where dissenting geneticists faced persecution (Soyfer, 1994).

## The Term ‘Epigenetics’ and Its Current Interpretation

The word ‘epigenetic’ was introduced by Conrad Hal Waddington, derived from the Aristotelian word *epigenesis* (Waddington, 1942). Initially, epigenetic was defined as the causal interactions between genes and their products which bring the phenotype into being. Waddington was interested in studying the processes of gene control during embryo development. In his experiments, he studied the crossveinless condition in the wing of *Drosophila melanogaster*, which can be produced by mutant alleles or simply by exposing pupae to heat shock. The capacity to respond to an external stimulus by some development reaction must itself be under genetic control. Waddington selected a fly population where most of the flies developed the crossveinless condition when exposed to a heat shock. After further selection, a considerable proportion of flies were crossveinless even in absence of heat. In another classical example, Waddington exposed Drosophila embryos to ether, which induces the phenotype bithorax in adult flies (four wings instead of two). After many generations of ether exposure and artificial selection for the bithorax phenotype, the selected flies expressed the aberrant phenotype even when they were not exposed to ether. This phenomenon is known as genetic assimilation (Waddington, 1953): an initially acquired trait becomes genetically stable within the population. The phenomenon of genetic assimilation is questioned as an example of Lamarckian inheritance. Rather, it is described as a genetic stabilization of a phenotype that was previously induced by some environmental stimulus. Under these scenarios, some genotypes determine a norm of reaction that varies with the environment (phenotypic plasticity). If an external stimulus breaks the current canalization, many aberrant individuals may be selected as parents of subsequent generations if selection for a specific trait favors alleles that determine the new phenotype. Then, as the frequency of these alleles increases, less environmental stimulus is required to produce a new phenotype.

At the time of Waddington’s experiments, genetics was an infant field, and little knowledge of the genome and its biology existed. The field of epigenetics has grown during the last decades, surely motivated by the increasing knowledge of DNA, molecular biology, gene regulation, and many related aspects (ENCODE Project Consortium, 2012). Nowadays, it is such a broad field that there is no consensus on the current definition of the term (Holliday, 2006; Bird, 2007; Deans and Maggert, 2015). Epigenetic includes any modification on the expression of genes that is due to factors other than a mutation in the DNA. This involves DNA methylation, post-translational modification of histones, regulation of gene expression by non-coding RNAs, and genome instabilities or any other force that could modify a phenotype.

Livestock genetics has also been impacted by the broad definition of epigenetics. Efforts to understanding epigenetics in livestock have focused mostly on the molecular epigenetic mechanisms that regulate the expression of certain genes or genomic regions, sometimes as a response to external environmental forces such as nutrition, maternal behavior, or climate changes. The debate on transgenerational epigenetic inheritance garners a smaller amount of scientific literature thus far, and possible strategies to use epigenetics (in a broad sense) in livestock populations have not yet been described (e.g., decreasing/increasing the frequency of un/favorable epigenetic marks or the use of epigenetic variation in genetic evaluations). The scarce amount of whole-genome epigenetic data and the high cost of screening the epigenotype of individuals are limiting factors to develop research on these aspects that involve large populations.

## Transgenerational Epigenetic Inheritance

There exists some controversy on the existence of a heritable component of epigenetics. It must be clarified that two types of epigenetic inheritance are usually referred to: (i) epigenetic marks, which can be inherited in the soma line as these marks are conserved during mitosis (Jablonka and Raz, 2009), and (ii) transgenerational epigenetic inheritance via the germ line, which controls patterns of gene expression that are passed from one generation to the next (Daxinger and Whitelaw, 2012). Some authors have proposed models to estimate the amount of epigenetic variance that is inherited in populations (Slatkin, 2009; Varona et al., 2015). However, it is often questioned that epigenetic marks are inherited in the germ line, at least in mammals, in which stable epigenetic marks have not yet been observed in more than three subsequent generations (Jablonka and Raz, 2009). Epigenetic marks in mammals are erased during meiosis and, therefore, not transmitted to progeny. Some epigenetic marks rarely escape this removal process and some epigenetic marks can occur *de novo*. Then, after fertilization and during pre-implantation of the early embryo, there is a second erasing stage: a wave of genome-wide demethylation that occurs rapidly in the paternal genome, except for centromeric regions, and relatively slower in the maternal genome (Jirtle and Skinner, 2007). Some of the epigenetic marks can, however, escape this reset stage. This is then followed by heavy *de novo* methylation, particularly in somatic lineages as these are established, that constitutes the genetic reprogramming of the cells in the embryo. In order for the epigenetic marks to be considered transgenerationally inherited in mammals, they should be conserved during more than three generations. Otherwise, differentiating them from the effects of the maternal environment on early ontogenesis is not straightforward. For instance, an external force that causes some kind of epigenetic mark (e.g., methylation at a certain gene regulatory region) may affect the pregnant mother, the fetus, and the germ cells of the fetus, but they disappear if the fourth generation is not exposed to such an environment. Further, imprinting (Khatib, 2012) is sometimes reported as some sort of epigenetic inheritance. Some epigenetic marks of the male germ line can escape the epigenetic reset and their effects may be observed in the progeny, but are erased in the germ line of the progeny and hence not transmitted to future generations (Whitelaw, 2015). Iqbal et al. (2015), in an experiment with mice, showed that although endocrine-disrupting chemicals exert direct epigenetic effects in exposed fetal germ cells, these marks are corrected by reprogramming events and are not transmitted to progeny. Recently, Varona et al. (2015) showed that no heritable epigenetic variance was captured in a large cattle population. Recent work from Martínez et al. (2014) in mice reported that in utero malnutrition results in epigenetic modifications in germ cells (F1) that are subsequently transmitted and maintained in somatic cells of the F2, although evidence about inheritance more than three generations still needs to be observed to support transgenerational inheritance. To the best of our knowledge there are no reported epigenetic marks transmitted via the male germ line during more than three generations.

Nonetheless, some epigenetic mechanisms might be controlled by heritable genetic effects. There are genotypes that are more susceptible to methylation than others (Coolen et al., 2011). Such as genotypes with a larger proportion of cytosines in the CpG islands that are more susceptible to methylation. Further, DNA codes for histone and DNA-folding proteins, which may also have epigenetic effects. These sorts of elements with potential epigenetic effects are heritable in an additive manner. The challenge here is how to separate them from additive (or epistasis or dominance) genetic effects and separate epigenetic from genetic variance (Hill et al., 2008). It could even be argued whether they should be treated differently at all from other genetic effects interacting in complex biologic systems.

Nevertheless, the most intriguing and promising aspect of the potential transgenerational role of epigenetics resides in cellular energetic status. Caloric energy is an important factor in the cellular environment that can influence cellular gene expression, DNA replication, growth, proliferation, differentiation, and even programmed death (Wallace et al., 2010). The high mutation rate of mitochondrial DNA (mtDNA) and its direct effect on bioenergetics make mtDNA an excellent genetic system for the adaptation of species subpopulations to regional differences in their energetic environment (Wallace, 2010). In fact, it has been proposed that epigenetic mechanisms that regulate expression of the nuclear genome influence mitochondria by modulating the expression of nuclear-encoded mitochondrial genes, and that a cell-specific mtDNA content (copy number) and mitochondrial activity can determine the methylation pattern of nuclear genes (Manev and Dzitoyeva, 2013). While most epigenetic marks are deleted during meiosis, mutations in the mtDNA may induce long-lasting epigenetic marks in genomic DNA. For example, some cancer studies have shown that mitochondrial dysfunction is associated with epigenetic alteration within the nuclear genome (Xie et al., 2007; Smiraglia et al., 2008). If these mutations were to occur in the gametes, then the influence of the environment on the adaptation of species through epigenetic mechanisms would be plausible. For instance, the ovary actively selects against those oocytes that contain lethal mutations in the mtDNA, but those that go unnoticed will not manifest in complex tissues and organs.

## Potential Uses of Epigenetics in Animal Breeding

Epigenetics is a recent research field, and to the best of our knowledge, is not yet used in selection or management strategies in livestock. Some insights, potential uses, and skepticisms have been previously proposed in cattle and sheep (González-Recio, 2012; Goddard and Whitelaw, 2014) and fish and molluscs (Moghadam et al., 2015). Although we share some skepticism about real implementation due to the high instability of epigenetic marks and the current cost of analyses, we next discuss the most relevant potential uses of epigenetics in livestock.

### Incorporating Epigenetic Information in Genomic Evaluations

The epigenotype of an individual controls the expression of the genotype. Distinguishing epigenetic effects, whether heritable or not, from heritable genetic effects would result in improved accuracy of prediction of breeding values (González-Recio, 2012). There are nonetheless some challenges to face in this implementation: statistical procedures to separate genetic from epigenetic variance must be developed (Varona et al., 2015) and most convenient tissues to analyze the epigenotype need to be determined for each trait, as epigenotypes differ from one tissue to the other, and even from cell to cell. This increases cost if the traits of interest are tissue-specific, as more than one methylation analysis per animal should be performed.

### Environment × Epigenotype × Genotype Studies

The environment can shape the epigenotype, which jointly with the genotype derive the phenotype. Understanding what external forces can model the epigenotype can help to design management strategies that promote certain epigenotypes. These favorable epigenotypes would promote the expression of productive traits in a more profitable fashion, such as improved disease resistance or increased longevity. Environment is especially important during embryo development, where genetic regulation occurs and can determine the adult life of the individual (Gluckman et al., 2008; Burdge et al., 2011; Bach, 2012; González-Recio et al., 2012). Proper nourishment and management of maternal and paternal environments that consider possible effects on the epigenome could produce healthier and more profitable progeny. In addition, if a given epigenetic status is known to have an effect on the phenotype, the epigenetic status itself could be treated as a phenotype for the prediction of future phenotypes as suggested by Goddard and Whitelaw (2014).

### Cross-breeding and Epigenetic Breeding Programs

If the epigenetic variance, or that due to imprinted genes, was sufficiently large, selection on male and female lines could be done separately (Goddard and Whitelaw, 2014). Mating programs could be designed considering the imprinting status of the progenitors to accommodate the most favorable epigenetic status to complement the breeding value. However, a low-cost procedure for epigenome screening would be necessary to implement these sorts of strategies.

## Final Remarks

In light of the state of the art, it is doubtful that a large amount of heritable epigenetic variance exists in livestock populations. Nonetheless, the epigenomic era brings exciting discoveries and challenges that can potentially be included in livestock breeding programs. Firstly, there is a need for a relatively inexpensive technology to sequence the epigenome on a large scale, as a large number of individuals are necessary to accurately estimate small epigenetic effects, and to estimate epigenetic variance at a population level. Secondly, statistical methods need to be developed to incorporate whole methylome information jointly with environment and massive DNA sequence information. Lastly, practical implementation must be carefully evaluated to successfully incorporate epigenetic information in livestock breeding. For instance, mating strategies to increase certain epigenotype frequencies are valid only if epigenetic marks are heritable. However, mate selection in order to obtain genotypes that favor a certain epigenotype could be implemented. Multidisciplinary genetic and management/nutrition practices can promote favorable epigenotypes in the populations, as well.

## Conflict of Interest Statement

The authors declare that the research was conducted in the absence of any commercial or financial relationships that could be construed as a potential conflict of interest.

## References

[B1] Bach A. (2012). Ruminant nutrition Symposium. Optimizing performance of the offspring: nourishing and managing the dam and postnatal calf for optimal lactation, reproduction, and immunity. *J. Anim. Sci.* 90 1835–1845. 10.2527/jas.2011-4516 21926322

[B2] Bird A. (2007). Perceptions of epigenetics. *Nature* 447 396–398. 10.1038/nature05913 17522671

[B3] Burdge G. C. Hoile S. P. Uller T. Thomas N. A. Gluckman P. D. Hanson M. A. (2011). Progressive, transgenerational changes in offspring phenotype and epigenotype following nutritional transition. *PLoS ONE* 6:e28282 10.1371/journal.pone.0028282 PMC3227644 22140567

[B4] Coolen M. W. Statham A. L. Qu W. Campbell M. J. Henders A. K. Montgomery G. W. (2011). Impact of the genome on the epigenome is manifested in DNA methylation patterns of imprinted regions in monozygotic and dizygotic twins. *PLoS ONE*:e25590 10.1371/journal.pone.0025590 PMC3184992 21991322

[B5] Darwin C. (1859). *On the Origin of Species.* London: John Murray.

[B6] Darwin C. (1868). *The Variation of Animals and Plants Under Domestication.* London: John Murray.

[B7] Daxinger L. Whitelaw E. (2012). Understanding transgenerational epigenetic inheritance via the gametes in mammals. *Nat. Rev. Genet.* 13 153–162. 10.1038/nrg3188 22290458

[B8] Deans C. Maggert K. A. (2015). What do you mean, “Epigenetic”? *Genetics* 199 887–896. 10.1534/genetics.114.173492 25855649 PMC4391566

[B9] Durrant A. (1962). The environmental induction of heritable change in Linum. *Heredity* 17 27–61. 10.1038/hdy.1962.2

[B10] ENCODE Project Consortium (2012). An integrated encyclopedia of DNA elements in the human genome. *Nature* 489 57–74. 10.1038/nature11247 22955616 PMC3439153

[B11] Gluckman P. D. Hanson M. A. Cooper C. Thornburg K. L. (2008). Effect of in utero and early-life conditions on adult health and disease. *N. Engl. J. Med.* 359 61–73. 10.1056/nejmra0708473 18596274 PMC3923653

[B12] Goddard M. E. Whitelaw E. (2014). The use of epigenetic phenomena for the improvement of sheep and cattle. *Front. Genet.* 5:247 10.3389/fgene.2014.00247 PMC4139735 25191337

[B13] González-Recio O. (2012). Epigenetics: a new challenge in the post-genomic era of livestock. *Front. Genet.* 2:106 10.3389/fgene.2011.00106 PMC3270332 22303400

[B14] González-Recio O. Ugarte E. Bach A. (2012). Trans-Generational effect of maternal lactation during pregnancy: a Holstein cow model. *PLoS ONE* 7:e51816 10.1371/journal.pone.0051816 PMC3527476 23284777

[B15] Hill W. G. Goddard M. E. Visscher P. M. (2008). Data and theory point to mainly additive genetic variance for complex traits. *PLoS Genet.* 4:e1000008 10.1371/journal.pgen.1000008 PMC2265475 18454194

[B16] Holliday R. (2006). Epigenetics: a Historical overview. *Epigenetics* 1 76–80. 10.4161/epi.1.2.2762 17998809

[B17] Iqbal K. Tran D. A. Li A. X. Warden C. Bai A. Y. Singh P. (2015). Epigenome reprogramming in the mammalian germline corrects deleterious effects of endocrine disruptors globally and at imprinted genes. *Genome Biol.* 16 59 10.1186/s13059-015-0619-z PMC4376074 25853433

[B18] Jablonka E. Raz G. (2009). Transgenerational epigenetic inheritance: prevalence, mechanisms, and implications for the study of heredity and evolution. *Q. Rev. Biol.* 84 131–176. 10.1086/598822 19606595

[B19] Jirtle R. L. Skinner M. K. (2007). Environmental epigenomics and disease susceptibility. *Nat. Rev. Genet.* 8 253–262. 10.1038/nrg2045 17363974 PMC5940010

[B20] Khatib H. (2012). *Livestock Epigenetics.* Edinburgh: Wiley-Blackwell.

[B21] Laforsch C. Tollrian R. (2004). Embryological aspects of inducible morphological defenses in Daphnia. *J. Morphol.* 262 701–707. 10.1002/jmor.10270 15487000

[B22] Lamarck J. B. (1809). *Zoological Philosophy: An Exposition with Regard to the Natural History of Animals*, trans. Elliot H. London: MacMillan, 107.

[B23] Manev H. Dzitoyeva S. (2013). Progress in mitochondrial epigenetics. *Biomol. Concepts* 4 381–389. 10.1515/bmc-2013-0005 25436587

[B24] Martínez D. Pentinat T. Ribó S. Daviaud D. Bloks V. W. Cebrià J. (2014). In utero undernutrition in male mice programs liver lipid metabolism in the second-generation offspring involving altered Lxra DNA methylation. *Cell Metab.* 19 941–951. 10.1016/j.cmet.2014.03.026 24794974

[B25] Moghadam H. Mørkøre T. Robinson N. (2015). Epigenetics—potential for programming fish for aquaculture? *J. Mar. Sci. Eng.* 3 175–192. 10.3390/jmse3020175

[B26] Slatkin M. (2009). Epigenetic inheritance and the missing heritability problem. *Genetices* 182 845–850. 10.1534/genetics.109.102798 PMC2710163 19416939

[B27] Smiraglia D. J. Kulawiec M. Bistulfi G. L. Gupta S. G. Singh K. K. (2008). A novel role for mitochondria in regulating epigenetic modification in the nucleus. *Cancer Biol. Ther.* 7 1182–1190. 10.4161/cbt.7.8.6215 18458531 PMC2639623

[B28] Sonneborn T. M. (1937). Sex, sex inheritance and sex determination in *Paramecium aurelia*. *Proc. Natl. Acad. Sci. U.S.A.* 23 378–385. 10.1073/pnas.23.7.378 16588168 PMC1076944

[B29] Soyfer V. N. (1994). *Lysenko and the Tragedy of Soviet Science.* New Brunswick: Rutgers University Press.

[B30] Steele E. J. Lindley R. A. Blanden R. V. (1998). *Lamarck’s Signature.* New York: Basic books.

[B31] Varona L. Munilla S. Mouresan E. F. Gonzalez-Rodriguez A. Moreno C. Altarriba J. (2015). A bayesian model for the analysis of transgenerational epigenetic variation. *G3 (Bethesda)* 5 477–485. 10.1534/g3.115.016725 25617408 PMC4390564

[B32] Waddington C. H. (1942). The epigenotype. *Endeavour* 1 18–20.

[B33] Waddington C. H. (1953). Genetic assimilation of an acquired character. *Evolution* 7 118–126. 10.2307/2405747

[B34] Wallace D. C. (2010). The epigenome and the mitochondrion: bioenergetics and the environment. *Genes Dev.* 24 1571–1573. 10.1101/gad.1960210 20679390 PMC2912552

[B35] Wallace D. C. Fan W. Procaccio V. (2010). Mitochondrial energetics and therapeutics. *Annu. Rev. Pathol.* 5 297–348. 10.1146/annurev.pathol.4.110807.092314 20078222 PMC3245719

[B36] Weismann A. (1904). *The Evolution Theory* Vol. 2 trans. Thomson J. A. Thomson M. R. London: Edward Arnold Publishers Ltd.

[B37] Whitelaw E. (2015). Disputing Lamarckian epigenetic inheritance in mammals. *Genome Biol.* 16 60 10.1186/s13059-015-0626-0 PMC4375926 25853737

[B38] Xie C. H. Naito A. Mizumachi T. Evans T. T. Douglas M. G. Cooney C. A. (2007). Mitochondrial regulation of cancer associated nuclear DNA methylation. *Biochem. Biophys. Res. Commun.* 364 656–661. 17964537 10.1016/j.bbrc.2007.10.047 PMC2443723

